# Exploring Contributing Factors of Solitary Drinking among Hong Kong Chinese Adolescents and Young Adults: A Descriptive Phenomenology

**DOI:** 10.3390/ijerph19148371

**Published:** 2022-07-08

**Authors:** Ka-Yan Ho, Katherine-Ka-Wai Lam, Cynthia-Sau-Ting Wu, Man-Nok Tong, Lai-Ngo Tang, Yim-Wah Mak

**Affiliations:** School of Nursing, Hong Kong Polytechnic University, Hong Kong SAR, China; kw-katherine.lam@polyu.edu.hk (K.-K.-W.L.); cynthia.wu@polyu.edu.hk (C.-S.-T.W.); man-nok.tong@polyu.edu.hk (M.-N.T.); lai-ngo.tang@polyu.edu.hk (L.-N.T.); yw.mak@polyu.edu.hk (Y.-W.M.)

**Keywords:** solitary drinking, alcohol use disorder, qualitative study, adolescent, young adult

## Abstract

Adolescents and young adults mostly drink alcohol because of social activities. However, some drink outside of normative social contexts, exhibiting a behaviour pattern known as solitary drinking. Increasing evidence indicates that solitary drinking is strongly associated with problematic drinking in adolescents and young adults. However, it remains unclear why individuals initiate and maintain this drinking habit. To address this gap in the existing literature, the current study explored the factors contributing to solitary drinking in this population. Descriptive phenomenology was used. A convenience sample of 44 solitary drinkers aged between 10 and 24 were invited to undergo individual semi-structured interviews. All interviews were audio-recorded and transcribed verbatim. The data were analysed by two researchers separately using Colaizzi’s method. Using qualitative descriptions, the following factors were identified as explaining the initiation and continuation of solitary drinking among adolescents and young adults: (1) enhancement and coping drinking motives, (2) social discomfort, (3) reduced self-control, (4) automatic mental process, and (5) a desperate response to stressors. Since reduced self-control plays an important role in long-term addiction, future studies should be conducted to determine potential applications of mindfulness-based interventions to improve self-control, which may prevent the progression from solitary drinking to alcohol use disorder.

## 1. Introduction

Alcohol is a group 1 carcinogen and represents the third leading cause of preventable death [1,2]. According to the National Institute on Alcohol Abuse and Alcoholism [3], drinking often begins during adolescence and continues into adulthood. Compared with other age groups, drinking is more prevalent in adolescence [3] and young adulthood [4]. A national survey in the US indicated that around 7 million young people aged 12–20 drank more than a few sips of alcohol in the past one month in 2019 [5]. Wu et al. [6] reported that approximately 19% of young adults aged 18–22 met the criteria for alcohol use disorder. In fact, adolescents and young adults are exposed to a greater risk of alcohol-related harm than other age groups because the brain continues to develop until the mid-twenties, hence drinking alcohol can lead to irreversible damage to the developing brain of adolescents and young adults [7].

According to the World Health Organization [8], adolescents are people aged 10–19 years, and young adults are those aged 20–24 years old. This population group was found to drink alcohol for a range of reasons [9,10]. Kuntsche et al. [9] reported that most young people drank alcohol as part of social activities. However, it was observed that some adolescents and young adults drink outside of normative social contexts, particularly drinking while alone in their daily life, exhibiting a pattern of behaviour that constitutes solitary drinking [10]. Although there are slight variations in definitions of solitary drinking used in previous studies, the term is generally defined as drinking without others present, or in the presence of non-interacting others [11]. Previous studies have reported that the prevalence rate of solitary drinking ranges from 15% to 24% among young adults [12,13,14]. However, a recent review indicated that the rate of solitary drinking increased to 40% among the high-risk group, especially among those in psychiatric- and addiction-treatment settings [11]. There is robust evidence that solitary drinking is associated with problems in the academic, legal, physical health, emotional, and social domains [15,16,17]. Importantly, an increasing number of studies have reported that solitary drinking is a risky drinking pattern among adolescents and young adults and is predictive of increased concurrent and prospective problematic drinking [11,12,18,19,20,21]. This notion is supported by the results of a recent meta-analysis of 21 studies involving 28,372 participants, which found moderate associations between solitary drinking, increased alcohol consumption, and drinking problems [11].

Despite the increasing number of studies reporting that solitary drinking is strongly associated with problematic drinking in adolescents and young adults [12,18,19,20,21], there is little evidence regarding the reasons for the initiation and continuation of this behaviour. To shed light on this under-researched area, the current study aimed to advance an area of the scientific literature on alcohol use by using a qualitative approach to summarize adolescents’ and young adults’ descriptions of their solitary drinking behaviours. Importantly, by examining these descriptions, we aimed to explore factors that contribute to solitary drinking in this population.

## 2. Materials and Methods

### 2.1. Design

Descriptive phenomenology was employed. This approach is commonly used to summarize people’s lived experience and enables researchers to obtain in-depth descriptions of factors contributing to the phenomenon (i.e., solitary drinking) [22]. We also took reference to previous descriptive phenomenological studies on addictive behaviours in terms of the procedures and analysis methods [23,24,25].

### 2.2. Respondents

To be eligible for the semi-structured interviews, respondents had to be 10–24 years old, able to speak Cantonese, and to self-report as exhibiting solitary drinking behaviour, which was defined as drinking while physically alone or drinking without interacting with other people [11].

### 2.3. Data Collection

Convenience sampling was used to recruit respondents. All respondents were recruited from the community via online advertisements or referrals from other non-governmental organizations. Due to the COVID-19 pandemic, respondents were given the option of conducting semi-structured interviews face-to-face or online. Before the interviews, all respondents were invited to complete a simple questionnaire to collect demographic information and alcohol-related characteristics, including the number of days on which they drank alcohol in the past 30 days, the number of drinks on each drinking day, the age at which they first drank alcohol, the percentage of time spent solitary drinking out of total drinking time, the number of solitary drinking episodes in the past 30 days, and alcohol dependence as assessed by the Alcohol Use Disorder Identification Test. The Alcohol Use Disorder Identification Test was developed by the World Health Organization as a self-reported instrument to identify unhealthy alcohol use. The Alcohol Use Disorder Identification Test scores range from 0 to 40. A score of 0 indicates no alcohol-related problems, a score of 1–7 indicates low-risk consumption, a score of 8–14 indicates harmful alcohol consumption, and a score of ≥15 indicates moderate-to-severe alcohol use disorder [26]. The interviews were conducted by two research assistants acting as an interviewer and an observer. The interviews followed a semi-structured interview guide covering five areas: (1) perception of solitary drinking, (2) perceived differences between solitary drinking and social drinking, (3) reasons for solitary and social drinking, (4) perceived role of solitary drinking in alcohol use disorder and (5) impacts of solitary drinking on their daily lives. Probing questions (e.g., “Why do you feel that way?” and “Can you give me some examples?”) were asked to solicit more information. The whole process was audio-recorded. Field notes that documented respondents’ facial expressions and gestures were recorded by the observer. Data collection ended upon data saturation, which was achieved after interviewing 44 respondents.

### 2.4. Data Analysis

Respondents’ demographic information and alcohol-related characteristics were summarized using descriptive statistics, including mean and standard deviation for continuous variables and frequency and percentage for categorical variables. The semi-structured interviews were transcribed verbatim, then the data were analysed separately by two researchers (KYH and KWKL). Analysis was conducted using Colaizzi’s method of descriptive phenomenology [27]. First, the two researchers repeatedly read the transcripts and listened to the audiotapes until they obtained a general understanding of the lived experience of the respondents. Meaningful units were extracted according to the research questions. The researchers would then compare the extracted meaningful units to identify similarities and discrepancies. On the basis of the similarities and discrepancies, these meaningful units were condensed into categories, clusters and themes, which were further organized into comprehensive descriptions. These descriptions were returned to the respondents for review and comment, ensuring that the descriptions were able to truly reflect their lived experience. Regular meetings were held among the research team (KYH; KKWL; CSTW; YWM) to resolve any disagreements raised during the data analysis.

### 2.5. Research Rigour

Research rigour can be safeguarded using multiple strategies [28]. Concerning transferability, detailed quotes and descriptions related to the lived experience of the respondents are presented in the results section. To ensure dependability and credibility, all of the interviews were conducted by the same research assistants. Triangulation was implemented, with one research assistant acting as an interviewer and another acting as an observer to collect non-verbal information, such as facial expressions and gestures of respondents, during information collection [29]. This non-verbal information was taken into consideration during the data analysis to supplement our understanding of the data and avoid misinterpretation [30]. The research assistants were reminded not to be judgemental and to allow the respondents to express themselves freely in the interviews. When respondents opted to have the interviews face-to-face, the interviews were arranged in a quiet interview room at a local university to avoid interruptions and protect confidentiality. Field notes were written down to document the details of the interviews, including date, time, venue, and key observations and conversations. All interviews were audio-recorded to avoid recall bias. To ensure that the codes and themes accurately reflected the lived experience of the respondents, we constantly compared the new data with existing data, as well as checking with the respondents. For confirmability, the two researchers (KYH and KWKL) who conducted the data analysis were asked to disregard their pre-existing beliefs and perceptions in relation to the topic, and to ensure that their judgements were not biased [28]. We also applied member checking, by which the respondents were invited to check the accuracy of the analysed data. Any inconsistencies or uncertainties were discussed and resolved in our regular research meetings.

### 2.6. Ethical Considerations

Ethical approval (HSEARS20201118001) was obtained from the institutional review board of Hong Kong Polytechnic University. This study (NCT05337488) was registered at clinicaltrial.gov. Throughout the study, we strictly adhered to principles of the Declaration of Helsinki (https://www.wma.net/policies-post/wma-declaration-of-helsinki-ethical-principles-for-medical-research-involving-human-subjects/, accessed on 1 June 2022) [31]. Prior to data collection, informed consent was sought from each respondent. The respondents received a comprehensive explanation of the study’s purpose and procedures. In addition, respondents were told that they could refuse to answer any questions in the interviews and withdraw from the study at any time without penalty. Although this study involved respondents below the age of 18 years, parental consent was not sought because drinking is a sensitive issue and respondents might not feel comfortable disclosing this information to their parents. All of the collected data were stored securely and could only be accessed by the project team.

## 3. Results

### 3.1. Demographic Characteristics of Respondents

We successfully achieved data saturation after recruiting and interviewing 44 respondents who were self-reported to exhibit solitary drinking behaviour. The current sample size was comparable to other descriptive phenomenological studies on addictive behaviours [23,24,25]. Of the respondents, 72.7% (n = 32) participated the interviews face-to-face, and the remaining opted to participate via online. Respondents’ mean age was 16.63 (SD = 2.61) years old and their mean household income was 2594.11 US dollars (SD = 735.23). Of the respondents, 72.7% (n = 32) were male, 43.2% (n = 19) attained upper secondary education, 27.3% (n = 12) were students, 27.3% (n = 12) were part-time employees, 63.6% (n = 28) lived in public rental housing and 84.1% (n = 37) were single. Regarding drinking behaviour, the mean age at which they first drank alcohol was 16.43 (SD = 2.61) years. On average, respondents reported drinking on 9.59 (SD = 7.45) of the past 30 days, and that they drank 2.57 (SD = 1.28) cans/glasses of alcoholic beverages on each drinking day. In addition, the respondents spent an average of 31.9% (SD = 16.15) of their drinking time engaged in solitary drinking and reported having an average of 4.55 (SD = 4.61) solitary drinking episodes in the past 1 month. Of the respondents, 27.3% (n = 12) were classified as exhibiting either harmful alcohol consumption or moderate-to-severe alcohol use disorder according to the Alcohol Use Disorder Identification Test results. Table 1 shows the details of demographic and alcohol-related characteristics of respondents.

### 3.2. Factors Contributing to Solitary Drinking Behaviours

Six themes were derived from the semi-structured interviews to illustrate the factors contributing to solitary drinking among Hong Kong Chinese adolescents and young adults. Table S1 shows the themes and examples of quotes from the interviews.

#### 3.2.1. Theme 1: Managing, Avoiding, or Escaping Negative Emotions (Coping Motive)

A number of respondents (n = 33) said they started and continued solitary drinking because it was a way for them to manage, avoid, or escape from negative emotions. Specifically, respondents (n = 21) mentioned that they were able to stop thinking about troubling matters after getting drunk. As indicated by one respondent (male, aged 15), “I drink alone when I am extremely stressed.” [Interviewer: “Why?”] “I always think about problems (while conscious). The thoughts repeat again and again. I feel annoyed. I would rather get drunk and go to sleep, so that I don’t have to think about it (troubling problem).” In addition, respondents (n = 33) reported that solitary drinking was different from social drinking: solitary drinking was associated with unhappiness, whereas social drinking was more associated with happy occasions. This was illustrated by one respondent as follows: “I mainly drink alone to deal with negative issues, such as having arguments with girlfriends, or being blamed by family members.” [Interviewer: “Do you think that drinking alone is different from social drinking?”]. “Yes, they’re different. I usually drink with friends to celebrate happy things. For example, I will drink at a birthday party. I will also drink with my friends at bars after dinner. Social drinking is positive.”

#### 3.2.2. Theme 2: Social Discomfort

Some respondents (n = 16) said they did not have a close friend they could speak with about their difficulties and began drinking alone when they were upset. This was illustrated by one respondent (male, aged 20), as follows: “Previously, I did talk to my friend about my problems. However, they were busy and not available all the time. I didn’t want to bother them. So, I started to drink alone.” Some respondents (n = 11) said they did have a friend they could speak to, but felt annoying doing so, especially when it involved something personal. Thus, they opted to drink alone when they were in a bad mood. As one respondent (male, aged 16) explained, “Sometimes, I ask my friends to accompany me, and I have to explain the details to them. This is annoying when I’m in a bad mood. Additionally, everyone has things that are private, which they don’t want to share. I prefer drinking alone.”

#### 3.2.3. Theme 3: Solitary Drinking Is the First Thought That Comes to Mind

A considerable number of respondents (n = 25) reported that they have previously adopted other methods, such as listening to music or sleeping, to cope with stress and negative feelings before they developed a solitary drinking habit. However, after engaging in such a habit for a certain period of time, they became heavily reliant on it and immediately thought about drinking when they were in a bad mood. Solitary drinking became a reflexive response to stress and negative feelings. This was illustrated by one respondent (male, aged 17) as follows: “When I am sad, I immediately think about alcohol.” [Interviewer: “Why?”] “Um … I think it’s the best way to escape.” [Interviewer: “Did you try other methods for handling your emotions?”] “No … I didn’t.” [Interviewer: “Why? Wouldn’t it be useful?”] “I don’t know how to explain it. When I’m stressed, drinking is the first thing that comes to my mind. It’s something automatic.”

#### 3.2.4. Theme 4: The Difficulties Cannot Be Solved

Some respondents (n = 19) reported that they proactively coped with the challenges using other constructive methods most of the time. However, they mentioned that not all of the difficulties they faced were resolvable. In such situations, they became desperate and felt like there was no way out, and hence solitary drinking was the only thing they could do to handle their emotions. This was further illustrated by one respondent (male, aged 23): “I am not the kind of person who likes turning to alcohol when facing difficulties. I just do that (solitary drinking) when difficulties cannot be resolved … Like having an argument with my girlfriend. My only choice is to drink alcohol, or we would break up. There’s nothing you can do to solve the problem … Friends also can’t help … Drinking is the only thing that I can do. At least I feel better when I drink.”

#### 3.2.5. Theme 5: Reduced Self-Control despite Understanding the Negative Consequences

Some respondents (n = 18) reported that they did not want to rely on solitary drinking to manage their stress and negative feelings because they thought that such behaviour was not good for their health, and sometimes led to bad consequences. As indicated by one respondent (male, aged 20) in the semi-structured interview, “I felt guilty for what I did when I was drunk (after solitary drinking). I remember a time when I called my girlfriend and argued with her. However, I couldn’t remember any details the next day. The worst thing was that I forgot to set the alarm and didn’t wake up for work.” Although the respondents (n = 16) were reluctant to engage in solitary drinking, they said that they had trouble controlling themselves when they were in a bad mood, ignored the negative health impacts, and continued their drinking habit. This was illustrated by one respondent (male, aged 24), as follows: “I always drink alone until I black out.” [Interviewer: “But you just told me that such a drinking habit is not good for you. Why do you continue?”] “Yes. I know that. But it’s hard to be so alert and think about the consequences when you’re extremely sad. I don’t want to give a thought to anything at that moment. When I’m fine (emotionally), I tell myself not to drink alone and not to drink so much next time. However, when I’m deeply sad, I ignore everything and lose control.”

#### 3.2.6. Theme 6: A Method of Relaxation (Enhancement Motive)

Although most respondents (n = 33) relied on solitary drinking as a method for coping with stress and negative feelings, some (n = 11) stated that they did it for relaxation. Specifically, these respondents (n = 11) felt that solitary drinking provided them with a break after having a long day at work, allowing them to recharge physically and mentally for the following workday. This was illustrated by one respondent (female, aged 20), as follows: “I like drinking alone because I feel relaxed afterwards.” [Interviewer: “Can you explain a little bit more?”] “… Um … (solitary drinking) is a way to recharge at the end of the day. I can listen to music while drinking. I like the atmosphere.” [Interviewer: “But why don’t you drink with your friends?”] “I want some personal time where I can sit down and do something I like. When I’m drinking with my friends, I still have to entertain them and talk to them. When I’m drinking alone, I can fully enjoy the time.” [Interviewer: “So, do you mean that you like drinking?”] “I don’t like drinking, but I like the feelings after drinking, and the atmosphere. I enjoy the feeling of getting tipsy.” [Interviewer: “Nothing to do with negative emotions?”] “Yes. It’s a way of recharging so I can handle work the next day.”

## 4. Discussion

Solitary drinking is a risky drinking pattern associated with increased alcohol consumption and frequent alcohol use. Previous literature indicates that solitary drinking is an early sign of alcohol use disorder [11]. Although an increasing number of quantitative studies have investigated the role of solitary drinking in harmful alcohol use, it remains unclear why adolescents and young adults initiate and continue solitary drinking. To shed light on this gap in the literature, the current qualitative study summarized respondents’ descriptions of such behaviours. Importantly, by examining descriptions, factors contributing to solitary drinking were identified.

In our semi-structured interviews, some respondents (n = 33) admitted that they started and continued solitary drinking because they relied on it as a method to cope with stress and negative feelings. In addition, these respondents (n = 33) reported that solitary drinking differed from social drinking, which was more related to the enhancement of positive emotions. These results are consistent with previous quantitative studies that identified a difference in drinking motivations between the two forms of drinking behaviours, with solitary drinking being driven by negative motivations and social drinking being driven by positive motivations [11,19,32,33]. Although a majority of our respondents (n = 33) identified negative motivations for solitary drinking, positive motivations were also reported by some respondents (n = 11). Some solitary drinkers preferred to drink alone because they wanted to have a short break from their everyday life to enjoy some quiet time and think about personal issues. Instead of being driven to drink alone by negative feelings, these solitary drinkers reported that solitary drinking was positive and provided relaxation. These qualitative findings are similar to a specific drinking pattern recently reported in Finland called pantsdrunk. Pantsdrunk is an asocial form of drinking culture in which a person consumes alcohol at home while largely undressed, without any intention to go out [34]. In accordance with our qualitative findings, Finns have been reported to consider this behaviour as a path to recovery and self-empowerment, to help them face future challenges [35]. In the current study, solitary drinking was found to be initiated and maintained because of both negative and positive motivations, suggesting that future studies should explore how this difference affects solitary drinking behaviours among adolescents and young adults, given that drinking motivation is well-established as a key factor influencing choices, patterns and outcomes regarding alcohol consumption [36].

The interview results revealed that some respondents (n = 25) had previously adopted other coping strategies, such as exercise and listening to music to manage their stress and negative feelings. However, once they developed the habit of solitary drinking, they gradually stopped using other coping strategies despite these strategies being useful, and mainly relied on solitary drinking to cope with stress and negative feelings because it was the first thing that came to mind. This phenomenon added to the previous literature that suggested that an automatic mental process can lead to uncontrolled use of alcohol [37]. The automatic mental process is similar to a reflexive action, generating an immediate thought that solitary drinking is essential in responding to stress and negative feelings. According to Bargh [36], automatic mental processes play an important role in substance abuse, constituting cognitions that develop through repeated use and reinforcing consequences, leading to instantaneous, habitual, and non-conscious responses (i.e., substance use). In an experimental study of 71 undergraduate students with various levels of drinking experience, Weingardt et al. [38] observed that alcohol-related concepts were automatically activated in memory among heavy drinkers when they were previously exposed to the positive outcomes of drinking. Weingardt et al. [38] also reported that repeated drinking experience coupled with positive outcomes increases the activation and accessibility of alcohol concepts in memory. As such, it is understandable that the behavioural decisions of our respondents might have been automatically biased in favour of solitary drinking instead of other activities when they reported multiple positive experiences (i.e., improved mood after solitary drinking).

In our semi-structured interviews, some respondents (n = 11) who reported the use of solitary drinking as a way to cope with or escape from their negative feelings admitted that they did not want to communicate about their problems with friends or family members. This finding suggests that social discomfort may be a contributing factor for solitary drinking among Hong Kong Chinese young adults. Our suggestion is indeed consistent with the results of a survey study in the US, which indicated that social discomfort moderated the relationship between negative feelings and solitary drinking [39]. Interestingly, most of our respondents were men. According to Yeung et al. [40], men are expected to play masculine roles in Chinese society, whereas sharing emotions implies weakness and vulnerability. In this cultural context, Chinese men may be less comfortable expressing their emotions, and may therefore adopt destructive self-reliant behaviours (e.g., solitary drinking to manage stress) [41].

The current results revealed that some respondents (n = 19) usually adopted constructive methods (e.g., seeking tangible and intangible support from friends and family members as coping strategies). However, when the problem was unresolvable, respondents became desperate and felt that solitary drinking was the only thing they could do. This phenomenon might be explained by the transactional model of stress and coping proposed by Lazarus and Folkman [42] who contended that coping responses are a consequence of interactions between a person and their environment. In addition, coping responses can be divided into two categories, including problem-focused coping, which leads to action to solve the causes of stress, and emotion-focused coping, which attempts to reduce negative emotions associated with stress. A previous study reported that when a person perceives an event as resolvable, they typically obtain more information to generate higher levels of problem-focused coping; however, when an event is regarded as unresolvable, a person will typically avoid taking constructive action, exhibiting higher levels of emotion-focused coping (e.g., solitary drinking to minimize their emotional reaction to the difficult situation) [42].

In our study, a proportion of respondents (n = 12) were classified as exhibiting either harmful alcohol consumption or a moderate or severe level of alcohol use disorder, assessed using the Alcohol Use Disorder Identification Test scores. This result is in accord with a previous report that solitary drinking is an early sign of alcohol use disorder [10]. However, according to our findings, not all solitary drinkers will progress to develop an alcohol use disorder. Some respondents (n = 11) reported that they perceived solitary drinking as a method of relaxation and have done so for several years. Other respondents (n = 21) reported that they had used solitary drinking to cope with negative feelings for a long time but were able to control themselves to avoid excessive drinking. Although we do not have a complete explanation for this phenomenon, drawing on previous studies on other addictive behaviours, including smoking and drug abuse [43,44], a reduced ability to control behaviour may be a factor leading to the escalation of drinking among solitary drinkers, eventually resulting in alcohol use disorder. This notion is supported by the relatively large number of respondents (n = 11) with moderate-to-severe alcohol use disorder mentioning that they thoroughly understood the negative consequences of continued solitary drinking but were unable to stay alert and control themselves when encountering particular stimuli (i.e., stress and negative feelings). A growing body of evidence suggests that reduced self-control plays an important role in long-term addiction and is strongly associated with the dysfunction of various neural circuits in the prefrontal cortex that are responsible for inhibitory control, including the anterior cingulate cortex, medial prefrontal cortex, and striatum [45,46,47]. Neuroimaging studies have reported that dysfunction in these two neural circuits increases vulnerability to smoking and drug abuse [45,48]. Currently, there is no conclusive aetiology of the dysfunction of the neural circuits. However, mindfulness-based interventions have been reported to have promising effects on strengthening the neural circuits mentioned above, potentially minimizing addiction [49]. Future studies should be conducted to explore the role of self-control in solitary drinking, and to determine the potential application of mindfulness-based interventions to improve self-control and prevent the progression from solitary drinking to alcohol use disorder.

### Limitations

The current study involved several limitations. First, all respondents were Hong Kong Chinese. Because cultural factors are known to affect drinking patterns, the current results may not be transferrable to other cultural contexts. Second, all respondents from this study were recruited from the community, rather than in hospital settings. Hence, we were unable to verify their self-reported drinking status (i.e., alcohol use disorder) using clinical records. Third, all recruited informants were given an option to have the interviews conducted via either face-to-face or online format due to the pandemic. Nevertheless, difference in data collection methods could lead to a bias in which our interviewer might not be able to clearly document and accurately respond to the informants’ non-verbal cues when conducting the interviews online. Therefore, the collected information might not be able to fully reflect the phenomenon we were investigating. Fourth, there were many uncontrolled variables, such as the individuals’ socioeconomic level and family history of alcohol use disorder that might affect the results. However, this study was a qualitative study which primarily focused on summarizing adolescents’ and young adults’ descriptions of their solitary drinking behaviours as a phenomenon. Importantly, by examining the qualitative descriptions, we aimed to explore factors that contribute to solitary drinking in this population using descriptive phenomenology. For descriptive phenomenology, it focuses on essences of phenomena that are present in lifeworld descriptions of any kind, such as interviews or written narratives [50]. Husserl, one of the influential scholars in qualitative studies, pointed out that phenomenon should be presented as perceived by the individual’s consciousness, instead of pursuing the objectivity [51]. Hence, in descriptive phenomenology, it is not common to control possible confounders in statistical analysis [50]. We understand that qualitative research is essential to exploring new phenomena that can later be tested quantitatively and this research is of value to the journal. We suggest that future studies examine the role of the identified factors in this qualitative study, including enhancement and coping drinking motives, social discomfort, reduced self-control, automatic mental processes, and desperate reactions to stressors using a quantitative approach, and determine how these factors relate to solitary drinking among adolescents and young adults by controlling the confounders, such as socioeconomic factors via different statistical methods, such as linear or logistic regression. This sequential approach is also commonly adopted in the field of addictive behaviours [23,24,25,52,53]. Fifth, age would influence our respondents’ responses in the semi-structured interviews. Particularly, adolescents might answer certain questions in a different way when compared to young adults. However, in this study, the majority of our respondents were young adults. Only less than 25% were adolescents. Given the small group of adolescents, sub-group analysis was not feasible in our current study. However, we suggest that future researchers conduct a subgroup analysis to explore whether there is any difference in the solitary drinking behaviours between adolescents and young adults. Sixth, convenience sampling was used for recruitment. However, this might lead to a bias by which the recruited informants were not chosen at random, hence the sample might be unlikely to represent the population being studied. This further limited the generalizability of our study findings.

## 5. Conclusions

This study bridges a gap in the existing literature by summarizing descriptions of solitary drinking among Hong Kong Chinese adolescents and young adults. Our findings revealed that enhancement and coping drinking motives, social discomfort, reduced self-control, automatic mental processes, and desperate reactions to stressors are possible factors contributing to the initiation and continuation of solitary drinking among this population. Future studies should be conducted to explore the possible application of mindfulness-based interventions in the prevention of alcohol use disorder among solitary drinkers.

## Figures and Tables

**Table 1:** Demographic and drinking characteristics of the respondents (n = 44).

Age, Mean (SD)	16.63 (2.61)
**Sex, n (%)**	
Male	32 (72.2)
Female	12 (27.3)
**Education attainment, n (%)**	
Primary or below	12 (27.3)
Secondary	19 (43.2)
Sub-degree or tertiary	13 (29.5)
**Marital status, n (%)**	
Single	37 (84.1)
Cohabiting	7 (15.9)
**Employment status, n (%)**	
Full-time employee	9 (20.5)
Part-time employee	12 (27.3)
Full-time student	12 (27.3)
Unemployed	11 (25.0)
**Types of housing, n (%)**	
Public rental housing	28 (63.6)
Private rental housing	11 (25.0)
Private housing (owner-occupier)	5 (11.4)
**Household income in US dollars, mean (SD)**	2594.11 (735.23)
**Number of solitary drinking episodes in the past 30 days, mean (SD)**	4.55 (4.61)
**Alcohol Dependence by the Alcohol Use Disorder Identification Test scores (0–40), n (%)**	
No problem from alcohol (0)	0 (0)
Low-risk consumption (1–7)	32 (72.7)
Harmful alcohol consumption (8–14)	8 (18.18)
Moderate-severe alcohol use disorder (≥15)	4 (9.10)

SD = Standard Deviation.

## Data Availability

Data will be available upon reasonable request.

## Supplementary Materials

The following is available online at https://www.mdpi.com/article/10.3390/ijerph19148371/s1, Table S1: An abridged table of themes and examples of quotes from the interviews.
