# Supplementary material for: Exploring Contributing Factors of Solitary Drinking among Hong Kong Chinese Adolescents and Young Adults: A Descriptive Phenomenology
Source: Int J Environ Res Public Health. 2022 Jul 8;19(14):8371. doi: 10.3390/ijerph19148371 (PMC9316069; doi:10.3390/ijerph19148371)
Supplement: Supplementary file 1 [file ijerph-19-08371-s001.zip › ijerph-1777425-SI.pdf]

Table S1. An abridged table of themes and examples of quotes from the interviews.

Factors contributing to solitary drinking	Theme	Examples of quotes
	1. Manage, avoid or escape negative emotion (coping motive)	I drink alone when I am extremely stressed…I always think about problems (while conscious). The thoughts repeat again and again. I feel annoyed. I would rather get drunk and go to sleep, so that I don't have to think about it (troubling problem)." (male, aged 15)
	2. Social discomfort	"Sometimes, I ask my friends to accompany me, and I have to explain the details to them. This is annoying when I'm in a bad mood. Also, everyone has things that are private, which they don't want to share. I prefer drinking alone." (male, aged 16)
	3. Solitary drinking is the first thing that appears in mind	"When I am sad, I immediately think about alcohol… I think it's the best way to escape…When I'm stressed, drinking is the first thing that comes to my mind. It's something automatic." (male, aged 17)
	4. The difficulties cannot be resolved	"I am not the kind of person who likes turning to alcohol when facing difficulties. I just do that (solitary drinking) when difficulties cannot be resolved … Like having an argument with my girlfriend. My only choice is to drink alcohol, or we would break up. There's nothing you can do to solve the problem … Friends also can't help … Drinking is the only thing that I can do. At least I feel better when I drink." (male, aged 23)
	5. Reduced self-control despite knowing the negative consequences	"I always drink alone until I black out… But it's hard to be so alert and think about the consequences when you're extremely sad. I don't want to give a thought to anything at that moment. When I'm fine (emotionally), I tell myself not to drink alone and not to drink so much next time. However, when I'm deeply sad, I ignore everything and lose control." (male, aged 24)

	6. A way for relaxation (enhancement motive)	“I like drinking alone because I feel relaxed afterwards… (solitary drinking) is a way to recharge at the end of the day. I can listen to music while drinking. I like the atmosphere…I want some personal time where I can sit down and do something I like. When I’m drinking with my friends, I still have to entertain them and talk to them. When I’m drinking alone, I can fully enjoy the time…I don’t like drinking, but I like the feelings after drinking, and the atmosphere. I enjoy the feeling of getting tipsy… It’s a way of recharging so I can handle work the next day.” (female, aged 20)
